# Imperatorin Influences Depressive-like Behaviors: A Preclinical Study on Behavioral and Neurochemical Sex Differences

**DOI:** 10.3390/molecules27041179

**Published:** 2022-02-10

**Authors:** Joanna Kowalczyk, Modestos Nakos-Bimpos, Alexia Polissidis, Christina Dalla, Nikolaos Kokras, Krystyna Skalicka-Woźniak, Barbara Budzyńska

**Affiliations:** 1 Independent Laboratory of Behavioral Studies, Medical University of Lublin, Jaczewskiego 4, 20-090 Lublin, Poland; joanna.kowalczyk19@gmail.com (J.K.); barbara.budzynska@umlub.pl (B.B.); 2 Center of Clinical, Experimental Surgery and Translational Research, Biomedical Research Foundation of the Academy of Athens, 11527 Athens, Greece; modestosbio@hotmail.com (M.N.-B.); apolissidis@bioacademy.gr (A.P.); 3 Department of Pharmacology, Medical School, National and Kapodistrian University of Athens, 11527 Athens, Greece; cdalla@med.uoa.gr (C.D.); nkokras@med.uo.gr (N.K.); 4 First Department of Psychiatry, Eginition Hospital, Medical School, National and Kapodistrian University of Athens, 11528 Athens, Greece; 5 Department of Natural Products Chemistry, Medical University of Lublin, 1 Chodźki Street, 20-093 Lublin, Poland

**Keywords:** depression, sex differences, imperatorin, furanocoumarins, serotonin, noradrenaline, forced swim test

## Abstract

Imperatorin, a naturally derived furanocoumarin, exerts promising neuropharmacological properties. Therefore, it might be applicable in the treatment of brain diseases such as depression. In the present project, we aimed to investigate the sex-dependent effects of imperatorin (1, 5, and 10 mg/kg) on behavior and neurochemistry associated with antidepressant effects. The depressive-like behaviors of male and female Swiss mice were investigated in a forced swim test (FST). Subsequently, High-Performance Liquid Chromatography (HPLC) was used to evaluate the level of serotonin, its metabolite, 5-HIAA, and noradrenaline, in mouse brains. The study revealed that only males responded to imperatorin (1 and 5 mg/kg) treatment and caused an antidepressant effect, such as with respect to depressive-like behaviors, lowering immobility time and increasing immobility latency. The HPLC analysis demonstrated that serotonin levels in the prefrontal cortex of females decreased with the middle dose of imperatorin (5 mg/kg), while in the male prefrontal cortex, the lower dose (1 mg/kg) boosted serotonin levels. There were no evident changes observed with respect to noradrenaline and serotonin metabolite levels in the male hippocampus. To conclude, we propose that imperatorin has antidepressant potential, seemingly only in males, influencing brain serotonin level, but the direct mechanism of action requires further investigation.

## 1. Introduction

Depression is a common psychiatric disorder characterized by a persistent feeling of despair along with an inability to experience pleasure [1]. Nowadays, depression is a leading cause of disability, and despite being a major contributing factor to the global burden of disease, current treatments face significant limitations, while elements of the etiopathology of the disease remain complex and elusive. Therefore, there is a need for personalized therapies and targeted medicines with higher and faster efficacy as well as fewer side effects.

Preclinical studies point to the beneficial effects of imperatorin (9-[(3-methyl-2-buten-1-yl)oxy]-7H-furo[3,2-g]chromen-7-one), a plant-derived secondary metabolite, in the treatment of mood-related diseases [2]. It was revealed both in vivo and in vitro studies that imperatorin could easily cross the blood-brain barrier (BBB), making it possible for therapeutic concentrations to reach the central nervous system (CNS) [3]. The work presented here stems from our previous research in which we showed procognitive (5 and 10 mg/kg), antioxidative (5 and 10 mg/kg) [4], anxiolytic (10 and 20 mg/kg; chronic treatment) [5,6] and anticonvulsant (40 mg/kg) [7], properties of imperatorin in mice. Imperatorin appears to act both as a GABA-A receptor activator (100 µM) [2] and as an acetylo- (AChEI) and butyrylcholinesterase inhibitor (BuChEI) and as a weak monoamine oxidase (MAO-A and MAO-B) inhibitor (30.0 and 24.84 µM, respectively) [8,9]. Additionally, in the antidepressant activity of imperatorin, the influence on MAO-A activity cannot be excluded [8]. Furthermore, imperatorin demonstrated anti-inflammatory activities by decreasing prostaglandin 2 and NO levels (1, 5, and 10 μg/mL) [10]. Finally, the repeated administration of 15 and 30 mg/kg imperatorin decreased immobility duration in prenatally stressed male offspring rats in a forced swim test (FST) and increased the levels of serotonin (5 hydroxytryptamine; (5-HT)) in both the hippocampus and frontal cortex [11]. Furthermore, 5-HT transporter levels were significantly decreased, and the concentration of the 5-HT_1A_ receptor increased in both structures [11]. However, the influence on depressive-like behaviors of imperatorin has yet to be compared in female and male animal models.

Depression is more common in women than men, and the analysis of sex differences in depression pathomechanisms and antidepressant response is imperative, in order to develop more effective treatment [12]. Indeed, evidence suggests that androgens and estrogens levels play a crucial role in mood disorders and depression [13,14,15,16]. In addition, there are neurobiological variabilities between the female and male hippocampus and prefrontal cortex. These variabilities can cause disparities in the course of depression, such as lower 5-HT neurotransmission, related to depression symptoms in women compared to men [17], lower expression of 5-HT_1A_ autoreceptors in the prefrontal cortex of depressed women [18], and a higher 5-HT_1A_ receptor binding ability in women compared to men [19]. Interestingly, untreated women patients suffering from depression had a higher level of 5-HT synthesis in the prefrontal cortex and regions of the limbic system compared to untreated men diagnosed with depression [20]. Moreover, studies suggest that female patients respond better to SSRIs than males [21,22,23]. Therefore, in the present study, we investigated the effects of imperatorin on the female and male behavioral response to the FST and the neurochemical mechanisms underlying this effect. Specifically, we evaluated 5-HT and noradrenaline (NA) levels in the hippocampus and prefrontal cortex after treatment with imperatorin and imipramine which served as a reference drug.

## 2. Results

### 2.1. Spontaneous Locomotor Activity in Female Mice under the Influence of Imperatorin (1, 5, 10 mg/kg) and Imipramine (30 mg/kg)

As seen in Table 1, the results confirmed that similar to male animals [4,5], imperatorin does not affect locomotor activity in female mice.

### 2.2. Imperatorin Decreases the Level of Depressive-like Behaviors in the Forced Swimming Test

**Figure 1:**
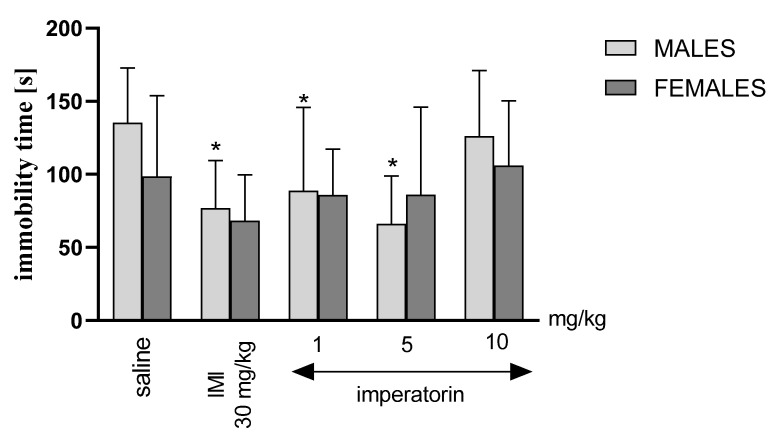
Duration of immobility in the FST in female mice. Saline, imipramine (IMI, 30 mg/kg), and imperatorin (1, 5, and 10 mg/kg) were injected (*i.p*.) 30 min before the test and immobility time [s] was measured; *n* = 8–10; * *p* < 0.05; mean +/− SD.

Two-way ANOVA revealed a statistically significant drug treatment effect (F _(4, 86)_ = 3.046, *p* = 0.0244) and no significant sex effect (F _(4, 86)_ = 0.7648, *p* = 0.3845), nor sex * drug interaction and sex * drug treatment differences (F _(4, 86)_ = 0.7385, *p* = 0.5698) in the FST immobility behavior duration. Bonferroni’s post hoc test showed that the immobility time was significantly decreased only in males after imperatorin treatment (* for 1 mg/kg: *p* < 0.05; 5 mg/kg: *p* < 0.05 vs. saline-treated control group) and imipramine (* for 30 mg/kg: *p* < 0.05 vs. saline-treated control group) treatment (Figure 1).

### 2.3. Immobility Latency in the Forced Swim Test 

**Figure 2:**
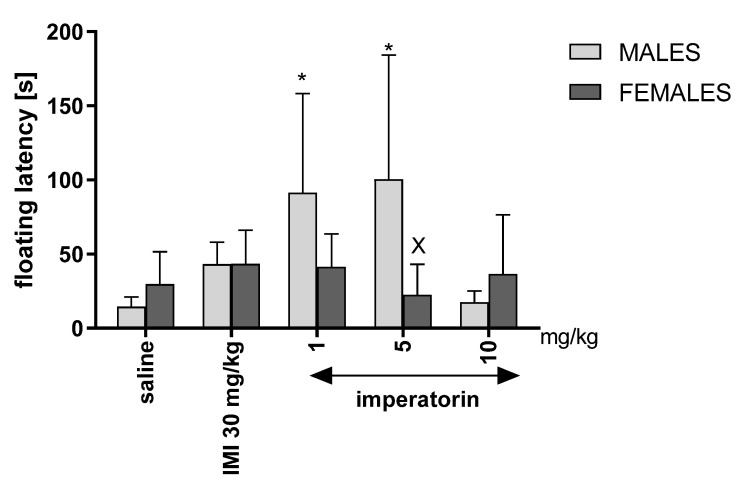
The immobility latency [s] as measured from the beginning of the FST to the first immobility event. Imipramine (IMI, 30 mg/kg, *i.p*.), imperatorin (1, 5, and 10 mg/kg*, i.p.*), and saline were administered 30 min before testing; *n* = 8–10; mean ± SD; * *p* < 0.05 vs. saline-treated male control group; ^X^ *p* < 0.05 vs. imperatorin-treated male group; Bonferroni’s test.

Two-way ANOVA revealed a statistically significant drug treatment effect (F _(4, 86)_ = 3.044, *p* = 0.0245) as well as sex * drug treatment differences (F _(4, 86)_ = 2.565, *p* = 0.0483), and no significant sex effect (F _(1, 86)_ = 1.291, *p* = 0.2608). Bonferroni’s post hoc test showed that the immobility latency increased significantly in males after 1 and 5 mg/kg imperatorin (* *p* < 0.05 vs. saline-treated control group) treatment (Figure 2).

### 2.4. Imperatorin Alters Serotonin Tissue Levels in the Hippocampus and Prefrontal Cortex

**Figure 3:**
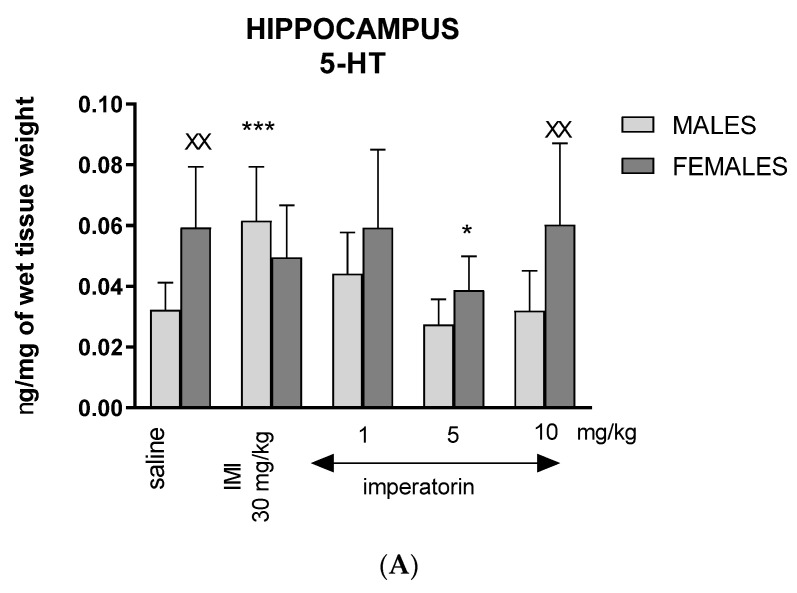

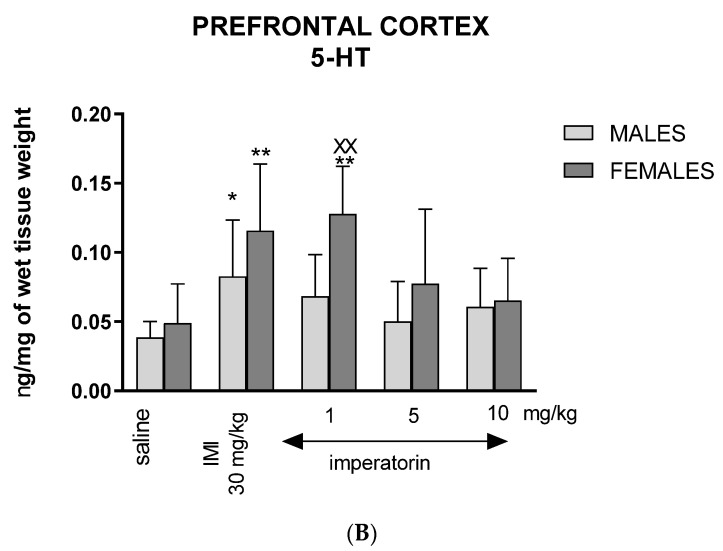
The Serotonin (5-HT) level in the hippocampus (**A**) and prefrontal cortex (**B**) after imipramine (IMI) and imperatorin injections (*i.p.*). Imipramine (IMI, 30 mg/kg) and imperatorin (1, 5 and 10 mg/kg) were administered 30 min before FST. The hippocampus (**A**) and prefrontal cortex (**B**) were dissected immediately after the test; *n* = 8–10; mean ± SD; *p < 0.05, ** *p* < 0.01, *** *p* < 0.001 vs. saline-treated control group, ^XX^ *p* < 0.01 vs. male group; Bonferroni’s test.

Two-way ANOVA analysis revealed a statistically significant sex difference (F _(1, 86)_ = 13.16, *p* = 0.0005), drug treatment effect (F _(4, 86)_ = 3.975, *p* = 0.0055), and significant sex * drug interaction (F _(4, 86)_ = 3.519, *p* = 0.0108) on hippocampal 5-HT levels (Figure 3A). Post hoc pairwise comparisons with Bonferroni’s correction showed that vehicle-treated females displayed higher hippocampal 5-HT tissue levels than males (*p* < 0.01). This sex difference was not observed following imipramine and imperatorin (1 mg/kg and 5 mg/kg) treatment but was evident following 10 mg/kg imperatorin treatment and in the control groups (^XX^ *p* < 0.01) for females vs. males. Moreover, in female mice, imperatorin (5 mg/kg) decreased 5-HT levels in comparison to vehicle-treated females: (* *p* < 0.05). In the male hippocampus, a significant increase in 5-HT levels was observed after imipramine injection (30 mg/kg; *** *p* < 0.001) vs. the male control group (Figure 3A).

Two-way ANOVA analysis revealed a statistically significant sex difference (F _(1, 86)_ = 11.85, *p* = 0.0009), and drug treatment effect (F _(4, 86)_ = 7.625, *p* < 0.0001), but no significant sex * drug interaction (F _(4, 86)_ = 1.531), *p* = 0.2016) on 5-HT levels in the prefrontal cortex (Figure 3B). Post hoc pairwise comparisons with Bonferroni’s correction showed increased level of 5-HT in females after imperatorin injection (1 mg/kg: ** *p* < 0.01 vs. saline). Imperatorin (1 mg/kg: ^XX^ *p* < 0.01) caused an increase in 5-HT levels in females vs., males. Additionally, imipramine (for 30 mg/kg: ** *p* < 0.01) in females and males (30 mg/kg; * *p* < 0.05) caused increase in 5-HT level vs. female and male control groups, respectively (Figure 3B). 

### 2.5. Imperatorin Alters 5-Hydroxyindoleacetic Acid Tissue Levels in the Hippocampus and Prefrontal Cortex 

**Figure 4:**
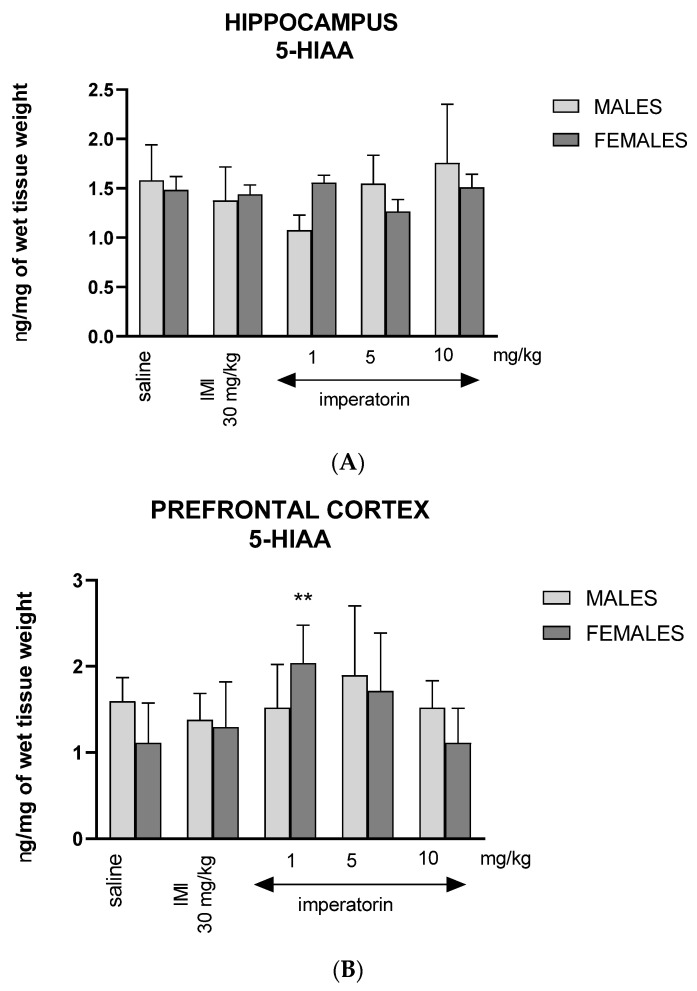
5-Hydroxyindoleacetic acid (5-HIAA) levels in the hippocampus (**A**) and prefrontal cortex (**B**) after imipramine (IMI) and imperatorin injections (*i.p.*) and comparison of sex differences. Imipramine (IMI, 30 mg/kg.) and imperatorin (1, 5, and 10 mg/kg.) were administered 30 min before the FST. The hippocampus (**A**) and prefrontal cortex (**B**) were dissected immediately after the test; *n* = 8–10; mean ± SD, ** *p* < 0.01 vs. female saline-treated group; Bonferroni’s test.

Two-way ANOVA analysis revealed no drug treatment effect (F _(4, 86)_ = 1.896, *p* = 0.1470, and no significant sex difference (F _(1, 86)_ = 0.04010, *p* = 0.8419), but a significant sex * drug interaction (F _(4, 86)_ = 3.020, *p* = 0.0234) in the hippocampus with respect to 5-HIAA levels was observed (Figure 4A).

Two-way ANOVA analysis revealed that there were statistically significant differences in the prefrontal cortex after drug administration (F _(4, 86)_ = 3.858, *p* = 0.0071) with no significant sex difference (F _(1, 86)_ = 1.273, *p* = 0.2632), and sex * drug interactions (F _(4, 86)_ = 2.448, *p* = 0.0548) (Figure 4B). Bonferroni’s post hoc test confirmed that imperatorin, at a dose of 1 mg/kg, increased 5-HIAA levels significantly in females (** *p =* 0.0029), compared to those treated with saline (Figure 4B).

### 2.6. Imperatorin Modifies Serotonin Turnover Ratio

**Figure 5:**
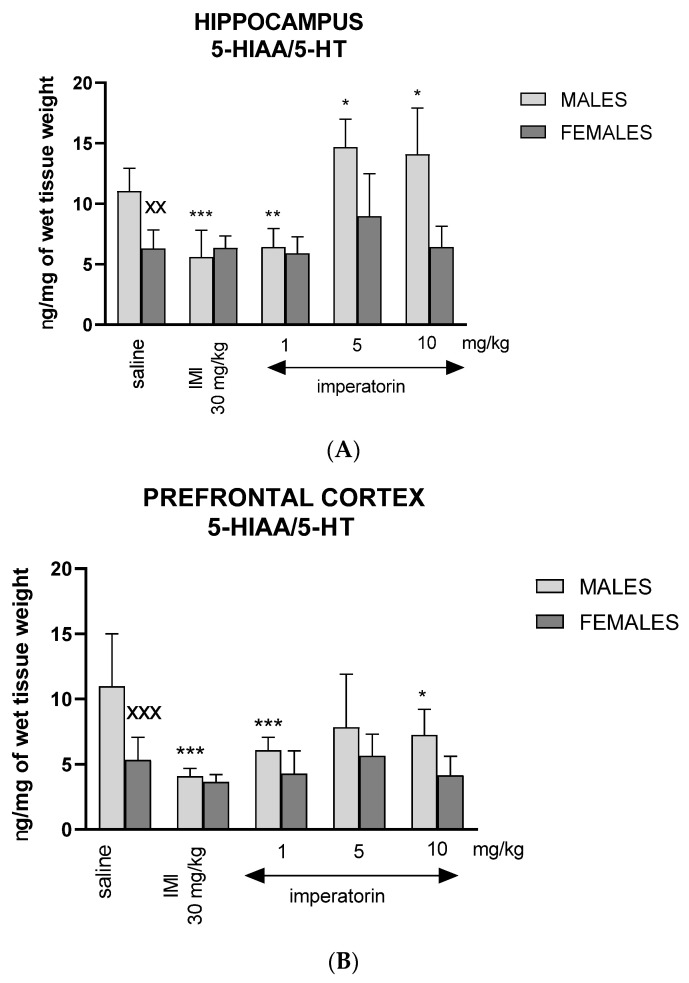
The 5-Hydroxyindoleacetic acid (5-HIAA)/serotonin (5-HT) ratio in the hippocampus (**A**) and prefrontal cortex (**B**) after imipramine (IMI) and imperatorin injections (*i.p.*). Imipramine (IMI, 30 mg/kg, *i.p.*) and imperatorin (1, 5, and 10 mg/kg, *i.p.*) were administered 30 min before the FST. The hippocampus (**A**) and prefrontal cortex (**B**) were dissected immediately after the test; *n* = 8–10; mean ± SD; * *p* < 0.05, ** *p* < 0.01, *** *p* < 0.001 vs. saline-treated control group, ^XX^ *p* < 0.01, ^XXX^ *p* < 0.001 vs. male group; Bonferroni’s test.

Two-way ANOVA showed a statistically significant effect of drug treatment (F _(4, 86)_ = 18.33, *p* < 0.0001), sex (F _(1, 86__)_ = 44.92, *p* < 0.0001), and a sex * drug interaction F _(4, 86)_ = 8.665, *p* < 0.0001), regarding the 5-HT turnover ratio (5-HIAA/5-HT ratio) in the hippocampus. Post hoc pairwise comparisons with Bonferroni’s correction revealed that imperatorin (1 mg/kg) and imipramine (30 mg/kg) significantly decreased hippocampal 5-HIAA/5-HT ratio in comparison to vehicle-treated males (** *p* < 0.01, *** *p* < 0.001, respectively). On the contrary, imperatorin (10 mg/kg and 5 mg/kg) significantly increased the 5-HIAA/5-HT ratio in male mice when compared to their vehicle-treated counterparts (* *p* < 0.001). The 5-HIAA/5-HT ratio was significantly lower in the female control group (^XX^ *p* < 0.01 vs. male saline-treated mice) (Figure 5A).

Two-way ANOVA showed a statistically significant effect of drug treatment (F _(4, 86)_ = 7.93, *p* = 0.0001), sex (F _(1, 86)_ = 28.22, *p* = 0.0001), and a sex * drug interaction (F _(4, 86)_ = 2.81, *p* = 0.0331) in the prefrontal cortex. Post hoc pairwise comparisons with Bonferroni’s correction revealed that imperatorin (1 mg/kg: *** *p* < 0.001; 10 mg/kg: * *p* < 0.01 vs. saline) and imipramine (30 mg/kg: *** *p* < 0.001) treatments decreased the 5-HIAA/5-HT ratio significantly in males. The 5-HIAA/5-HT ratio was significantly lower in the female control group (^XXX^ *p* < 0.001 vs. male saline-treated mice) (Figure 5B). 

### 2.7. Imperatorin Influences Noradrenaline Tissue Levels 

**Figure 6:**
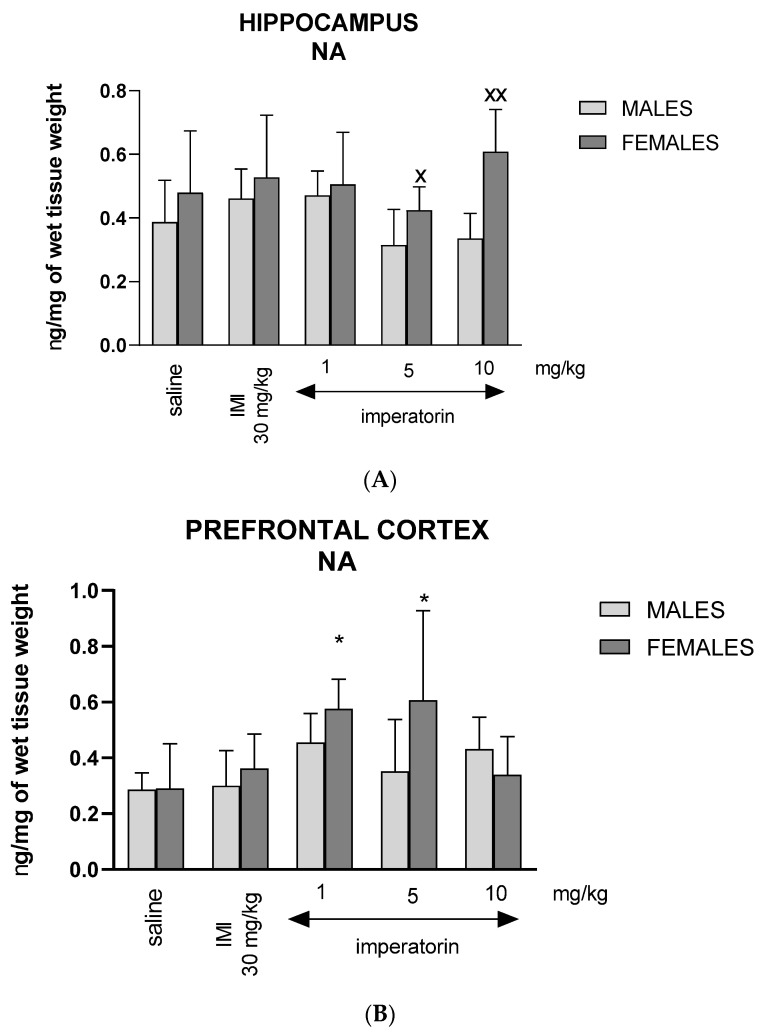
The level of NA (ng/mg) in the (**A**) hippocampus and (**B**) prefrontal cortex after imperatorin injection (1, 5, and 10 mg/kg). *n* = 8–10; mean ± SD; * *p* < 0.05, vs. saline-treated control group, ^X^ *p* < 0.05, ^XX^*p* < 0.01 vs. male group; Bonferroni’s test.

Two-way ANOVA revealed a statistically significant sex effect (F _(1, 86)_ = 12.41, *p* = 0.0008), with no drug treatment effect (F _(4, 86)_ = 1.99*, p* < 0.1076), and no significant sex * drug interaction (F _(4, 86)_ = 1.58, *p* = 0.1913) on hippocampal NA levels (Figure 6A). Post hoc pairwise comparisons with Bonferroni’s correction revealed increased NA hippocampal levels in females following imperatorin treatment (5 mg/kg: ^X^ *p* < 0.05; 10 mg/kg: ^XX^ *p* < 0.01 vs. males) (Figure 6A).

Two-way ANOVA analysis showed a statistically significant drug treatment effect (F _(4, 86)_ = 4.28, *p* = 0.0043) with no statistically significant influence of sex * drug interaction (F _(4, 86)_ 2.06*, p* = 0.0978, nor a sex main effect (F _(1, 86)_ = 2.88, *p* = 0.0951) in the prefrontal cortex on NA levels (Figure 6B). Post hoc pairwise comparisons with Bonferroni’s correction showed an increased NA level in females following the administration of imperatorin (1 and 5 mg/kg: * p < 0.05 vs. saline) (Figure 6B).

## 3. Discussion

The current project investigated the potential influence of imperatorin on depressive-like behaviors in the FST and associated neurochemical changes in both sexes. There were perceivable alterations in the animals’ behavioral response following imperatorin treatment, supported by variations in 5-HT and NA levels. In addition, the decrease in immobility duration following imperatorin application (1 and 5 mg/kg) was evident in males but not in females, as shown for the first time in this study (Figure 1).

Indeed, the present results showed that imperatorin (1 mg/kg and 5 mg/kg) and imipramine (30 mg/kg) influence depressive-like behaviors only in male not in female Swiss mice. Regarding imipramine, our findings are in line with a previous study conducted by Carrier and Kabbaj, where the social isolation of rats caused anxiolytic and antidepressant effects of imipramine only in males [24]. Collectively, our findings suggest that males responded more effectively to imperatorin treatment than females as depressive-like behavioral indices were lower (i.e., immobility time) and appeared later (i.e., immobility latency) than the saline male control group.

Moreover, imperatorin (1, 5, and 10 mg/kg) exerted a perceivable dose-dependent effect on depressive-like behaviors in male mice, whereas, as mentioned, no such effect was observed in female mice. The weaker response to the highest dose of imperatorin might be caused by the liver enzymes’ saturation that turns imperatorin into two active metabolites, xanthotoxol and heraclenin [25]. The pharmacological effects of the active metabolites were evaluated in several previous studies. Xanthotoxol showed neuroprotective anti-inflammatory properties and a blockage of the calcium channels [26,27]. On the other hand, heraclenin, the second active metabolite of imperatorin, was also found to possess anti-inflammatory properties [28]. Thus, we may hypothesize that the activity of imperatorin is linked to its metabolites, xanthotoxol and heraclenin, a decreased immobility duration, and elongated immobility latency, although further studies are required to explain this mechanism.

It is also of interest that imperatorin did not decrease the immobility time in the FST of females. A similar pattern, with males but not females responding to the treatment, was recently observed by our group after treatment with another furanocoumarin, xanthotoxin [29]. In the current study, as in the previous study with xanthotoxin [29], at baseline, male vehicle-treated animals had a slightly, but not statistically significant, longer immobility duration than females. Several conflicting results have been reported regarding baseline sex differences in the FST [30]. However, the behavioral response to the treatment with coumarins and the typical decrease in immobility time in the FST appears to be more prominent in males than in females. Such findings are in agreement with clinical evidence revealing that men respond faster and more favorably to imipramine than women [31]. Thus, our study corroborates that imipramine and, importantly, imperatorin-derived antidepressants may not be optimal for treating depression in women.

Our results showed that the acute administration of imperatorin influenced tissue levels of 5-HT only in the female hippocampus. Additionally, only the 1 mg/kg dose of imperatorin increased 5-HT tissue levels in the female prefrontal cortex, although no behavioral effects were observed. Concerning the 5-HT turnover ratio (5-HIAA/5-HT), it is evident that males displayed a more extensive release and/or metabolism of 5-HT than females in both the prefrontal cortex and hippocampus. Imipramine (30 mg/kg) reduced the sex difference in 5-HT turnover in both brain structures. All doses of imperatorin in the prefrontal cortex decreased 5-HT turnover in males. However, it was still higher than in females, where 5-HT release and/or metabolism stayed unchanged, regardless of the applied dose compared to the vehicle-treated control group. On the contrary, in the hippocampus, doses of 5 and 10 mg/kg of imperatorin increased the speed of 5-HT release and/or metabolism . The different tendencies of changes in 5-HT levels after imperatorin and xanthotoxin injections may result from the disparity in mechanisms of action, drug metabolism, and/or the different pharmacological activity of active metabolites. Indeed, substantial evidence shows significant sex differences in the pharmacokinetics of antidepressants [21,22,23,30,31,32]. This is a limitation of the present study that requires further experiments to examine the level of active metabolites and their activity in the brain. Based on current data, we can speculate that the pharmacokinetics of imperatorin in female and male mice are also differentiated and cause discrepancies in the observed behavioral and neurochemical effects.

Interestingly, a correlation exists between the behavior of animals in the FST and the level of the 5-HT turnover ratio in the prefrontal cortex [32]. The lower 5-HT turnover ratio in the prefrontal cortex but not in the hippocampus correlates well with the lower immobility time in the FST. Clinical studies revealed that brain 5-HT turnover was significantly elevated in unmedicated patients with major depressive disorder, compared with healthy subjects. Following effective therapy with an antidepressant drug, brain 5-HT turnover was reduced [33]. Thus, we may conclude that a reduced 5-HT turnover ratio is a marker of the antidepressant efficacy of imipramine in male mice tested in our study. 

Regarding NA, the only doses of imperatorin that increased the NA tissue levels were 1 and 5 mg/kg in the female, but not in the male prefrontal cortex. This finding does not correlate well with the behavioral response and perhaps it further corroborates that female neurotransmitter systems are more sensitive to change following pharmacological manipulations. There was no influence on the NA tissue levels in the hippocampus, so the hippocampus might not be engaged in the imperatorin’s mechanism of action.

A recent in vitro study showed that imperatorin could have an affinity to the 5-HT_7_ receptor, which is involved in the neurobiology of depression and its pharmacological antagonism decreased immobility in the FST [34], Therefore, it is possible that the influence on depressive-like behaviors observed in males could be related to the effect of imperatorin on 5-HT receptors without necessarily affecting the neurotransmitter levels. The monoamine theory of depression states that alongside 5-HT and NA, dopamine might also be involved in disease development and treatment response [35]. Based on this theory, another explanation could be that imperatorin targets the adenosine and dopamine systems. Indeed, the structure-based optimization study on synthetic coumarin 3-(4-Bromophenyl)-8-hydroxycoumarin revealed high antagonistic potency and selectivity to the A3 receptor [36]. There are also findings demonstrating that adenosine receptors are colocalized with dopamine receptors, thus influencing their activity [37]. Since dopamine D2 receptor activity is usually inhibited by adenosine A2A receptors co-localized on the same population of striatal neurons adenosine may play a crucial role in dopamine signaling modulation. Additionally, adenosine A2A receptors and dopamine D4 receptors are expressed in the striatal and cortical areas, respectively, which are known to be involved in depression [38]. An investigation of the dopaminergic system represents an interesting avenue for future research into imperatorin’s mechanism of action.

## 4. Materials and Methods

### 4.1. Drugs

**Figure 7:**
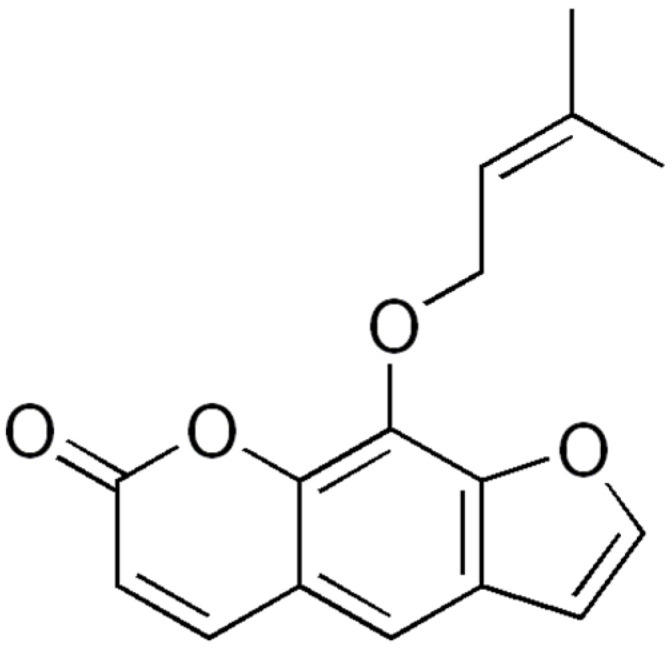
The structure of imperatorin (9-(3-methylbut-2-enoxy)furo[3,2-g]chromen-7-one).

Upon isolation [5], imperatorin [9-(3-methylbut-2-enoxy)furo[3,2-g]chromen-7-one (IUPAC name)] (Figure 7) was suspended in Tween 80 (Sigma, St. Louis, MO, USA), and dissolved in saline (0.9% NaCl). Imperatorin was injected intraperitoneally (*i.p.*) at a volume of 10 mL/kg of body weight. Based on our previous studies, 1, 5, and 10 mg/kg imperatorin doses were selected [4,5,6]. Imipramine (Sigma, St. Louis, MO, USA) was dissolved in saline (0.9% NaCl) and injected (*i.p.*) at a dose of 30 mg/kg [4,5,6]. All solutions were prepared fresh immediately before injections.

### 4.2. Animals

Two-month-old Swiss mice of both sexes, males (*n* = 50) and females (*n* = 50), 20–25 g, were group-housed under the conditions determined by the Experimental Medicine Center in Lublin. Rights and permission of the Local Ethical Committee on Animal Testing (permission number 2/2019; 51/2020) were obtained. Light cycle (12 h light/12 h dark), temperature (22 °C), and humidity conditions (55%) were stable throughout experimentation. Mice were divided into 10 treatment groups, 5 of which were male (*n* = 10/group) and 5 of which were female (*n* = 10/group). All females were in different phases of the estrous cycle because of the randomization rule in order to avoid bias in the study. In addition, mice were randomly assigned to different treatment groups, and the experimenter was blinded to the allocation when conducting the FST and Spontaneous Locomotor Activity test and for the outcome assessment, according to *ARRIVE* guidelines.

### 4.3. Spontaneous Locomotor Activity

Previous studies with imperatorin conducted by our group [4,5,6] revealed that imperatorin (1–10 mg/kg) does not alter the locomotor activity of male mice. As imperatorin has not been studied in females, we performed a pilot experiment on imperatorin’s effects on female locomotor activity. Female mice were randomly allocated to receive either saline, imperatorin (1, 5, and 10 mg/kg), or imipramine (30 mg/kg). The test was performed immediately following injections in the Columbus Instruments Opto-Varimex-4 Auto-Track (transparent cages: 43 × 43 × 32 cm^3^, two rows of photocells), as previously described [29]. The examination lasted 30 min and the distance traveled was measured. 

### 4.4. Forced Swim Test (FST)

The FST was performed 30 min after acute drug administration, and the duration of immobility behavior, as well as the immobility latency, were measured. The procedure was performed and recorded as previously described [29]. The recordings were analyzed using the open-source software Kinoscope [39].

### 4.5. Neurochemical Assays

To estimate neurotransmitters concentration in the brain, mice were sacrificed immediately after the FST by decapitation. The hippocampi and prefrontal cortices were dissected on ice, rinsed with saline, weighed, and snap-frozen on dry ice. Samples were stored at −80 °C until analysis. The homogenization of dissected tissues was performed as previously described [40]. Reverse phase ion-pair chromatography on an isocratic pump (YL9112 Instrument Co., Ltd., Gyeonggi-do, Korea) was used together with an electrochemical detector (BASi LC-EC, Bioanalytical Systems, Inc., Indianapolis, IN, USA) to determine tissue monoamine levels, as previously described, [40]. In addition to the assay of 5-HT and 5-HIAA tissue levels, the 5-HT turnover ratio was also calculated (5-HIAA/5-HT ratio). This index corresponds to the serotonergic activity being better than 5-HT or 5-HIAA tissue levels alone because it reflects 5-HT release and/or metabolic activity as described extensively elsewhere [41,42,43]. The results are expressed as ng/mg of wet tissue weight.

### 4.6. Statistical Analysis

Results were analyzed using a two-way ANOVA comparing sex (female vs. male) and drug treatment (control vs. imperatorin/imipramine), followed by post hoc multiple pairwise comparisons with Bonferroni’s correction using GraphPad Prism version 8. Statistical significance was indicated when *p* < 0.05 and results are expressed as mean ± SD.

## 5. Conclusions

To conclude, in our study, despite the observed antidepressant effect in the FST following imperatorin administration in males, the mobilization of the serotoninergic system was not consistent with the behavioral response. Several changes were observed in the female brain neurochemistry in the absence of a related behavioral response. Thus, it may be suggested that the serotoninergic and noradrenergic mechanisms are not crucial for the observed decrease in depressive-like behaviors caused by imperatorin, and it is necessary to look for other possible pathways. Additionally, we are aware of the study’s limitations regarding the duration of the drug administration. Typical therapies of depression last at least several weeks. Thus, another step of our research will be a long-term administration of imperatorin to establish its antidepressant effects.

## Figures and Tables

**Table 1:** Imperatorin (1, 5, and 10 mg/kg) does not affect spontaneous locomotor activity (30 min) in female Swiss mice. Mice were placed in actimeters immediately after saline, imperatorin (IMP 1, 5, and 10 mg/kg, *i.p*.), or imipramine (IMI 30 mg/kg, *i.p*.) injections. Data are presented as mean ± SD. *n* = 8; *p* < 0.05 vs. saline-treated female control group, one-way ANOVA: Tukey’s test.

MEANS ± SD Photocell Beam Breaks /30 min					
	Saline	IMI 30 mg/kg	IMP 1 mg/kg	IMP 5 mg/kg	IMP 10 mg/kg
Females	7734 ± 3618	6623 ± 5156	10,242 ± 7766	7390 ± 3906	7960 ± 4662

## Data Availability

Data available on request from the authors.

## References

[1] Wang J., Wu X., Lai W., Long E., Zhang X., Li W., Zhu Y., Chen C., Zhong X., Liu Z. (2017). Prevalence of depression and depressive symptoms among outpatients: A systematic review and meta-analysis. BMJ Open.

[2] Kozioł E., Skalicka-Woźniak K. (2016). Imperatorin-pharmacological meaning and analytical clues: Profound investigation. Phytochem. Rev..

[3] Tun T., Kang Y.S. (2017). Imperatorin is Transported through Blood-Brain Barrier by Carrier-Mediated Transporters. Biomol. Ther..

[4] Budzynska B., Boguszewska-Czubara A., Kruk-Slomka M., Skalicka-Wozniak K., Michalak A., Musik I., Biala G. (2015). Effects of imperatorin on scopolamine-induced cognitive impairment and oxidative stress in mice. Psychopharmacology.

[5] Budzynska B., Kruk-Slomka M., Skalicka-Wozniak K., Biala G., Glowniak K. (2012). The effects of imperatorin on anxiety and memory-related behavior in male Swiss mice. Exp. Clin. Psychopharmacol..

[6] Budzynska B., Boguszewska-Czubara A., Kruk-Slomka M., Skalicka-Wozniak K., Michalak A., Musik I., Biala G., Glowniak K. (2013). Effects of imperatorin on nicotine-induced anxiety- and memory-related responses and oxidative stress in mice. Physiol. Behav..

[7] Luszczki J.J., Glowniak K., Czuczwar S.J. (2007). Imperatorin enhances the protective activity of conventional antiepileptic drugs against maximal electroshock-induced seizures in mice. Eur. J. Pharmacol..

[8] Shaymaa M.M., Narayan D.C., Nesma M.M., Soad A.L.B., Babu L.T., Samir A.R. (2020). Promising selective MAO-B inhibition by sesamin, a lignan from Zanthoxylum flavum stems. Saudi. Pharm. J..

[9] Granica S., Kiss A.K., Jarończyk M., Maurin J.K., Mazurek A.P., Czarnocki Z. (2013). Synthesis of imperatorin analogs and their evaluation as acetylcholinesterase and butyrylcholinesterase inhibitors. Arch. Pharm..

[10] Huang G.J., Deng J.S., Liao J.C., Hou W.C., Wang S.Y., Sung P.J., Kuo Y.H. (2012). Inducible nitric oxide synthase and cyclooxygenase-2 participate in anti-inflammatory activity of imperatorin from Glehnia littoralis. J. Agric. Food Chem..

[11] Cao Y., Liu J., Wang Q., Liu M., Cheng Y., Zhang X., Lin T., Zhu Z. (2017). Antidepressive-like effect of imperatorin from Angelica dahurica in prenatally stressed offspring rats through 5-hydroxytryptamine system. Neuroreport.

[12] Balta G., Dalla C., Kokras N. (2019). Women’s Psychiatry. Adv. Exp. Med. Biol..

[13] Zarrouf F.A., Artz S., Griffith J., Sirbu C., Kommor M. (2009). Testosterone and depression: Systematic review and meta-analysis. J. Psychiatr. Pract..

[14] Solomon M.B., Herman J.P. (2009). Sex differences in psychopathology: Of gonads, adrenals and mental illness. Physiol. Behav..

[15] Parker G.B., Brotchie H.L. (2004). From diathesis to dimorphism: The biology of gender differences in depression. J. Nerv. Ment. Dis..

[16] Ahokas A., Kaukoranta J., Wahlbeck K., Aito M. (2001). Estrogen deficiency in severe postpartum depression: Successful treatment with sublingual physiologic 17beta-estradiol: A preliminary study. J. Clin. Psychiatry.

[17] Moreno F.A., McGahuey C.A., Freeman M.P., Delgado P.L. (2006). Sex differences in depressive response during monoamine depletions in remitted depressive subjects. J. Clin. Psychiatry.

[18] Szewczyk B., Albert P.R., Burns A.M., Czesak M., Overholser J.C., Jurjus G.J., Meltzer H.Y., Konick L.C., Dieter L., Herbst N. (2009). Gender-specific decrease in NUDR and 5-HT1A receptor proteins in the prefrontal cortex of subjects with major depressive disorder. Int. J. Neuropsychopharmacol..

[19] Mann J.J., Brent D.A., Arango V. (2001). The neurobiology and genetics of suicide and attempted suicide: A focus on the serotonergic system. Neuropsychopharmacology.

[20] Frey B.N., Skelin I., Sakai Y., Nishikawa M., Diksic M. (2010). Gender differences in alpha-[(11)C]MTrp brain trapping, an index of serotonin synthesis, in medication-free individuals with major depressive disorder: A positron emission tomography study. Psychiatry Res..

[21] Khan A., Brodhead A.E., Schwartz K.A., Kolts R.L., Brown W.A. (2005). Sex differences in antidepressant response in recent antidepressant clinical trials. J. Clin. Psychopharmacol..

[22] Joyce P.R., Mulder R.T., Luty S.E., McKenzie J.M., Rae A.M. (2003). A differential response to nortriptyline and fluoxetine in melancholic depression: The importance of age and gender. Acta Psychiatr. Scand..

[23] Kornstein S.G., Schatzberg A.F., Thase M.E., Yonkers K.A., McCullough J.P., Keitner G.I., Gelenberg A.J., Davis S.M., Harrison W.M., Keller M.B. (2000). Gender differences in treatment response to sertraline versus imipramine in chronic depression. Am. J. Psychiatry.

[24] Carrier N., Kabbaj M. (2012). Testosterone and imipramine have antidepressant effects in socially isolated male but not female rats. Horm. Behav..

[25] Zhao G., Peng C., Du W., Wang S. (2014). Simultaneous determination of imperatorin and its metabolites in vitro and in vivo by a GC-MS method: Application to a bioavailability and protein binding ability study in rat plasma. Biomed. Chromatogr..

[26] He W., Chen W., Zhou Y., Tian Y., Liao F. (2013). Xanthotoxol exerts neuroprotective effects via suppression of the inflammatory response in a rat model of focal cerebral ischemia. Cell. Mol. Neurobiol..

[27] Liu J., Lian Q., Zhou L., Zhou Q., He W., Zhu Z., Lai F. (2005). Calcium antagonistic effect of Xanthotoxol on isolated guinea pig atria. Zhong Yao Cai.

[28] Márquez N., Sancho R., Ballero M., Bremner P., Appendino G., Fiebich B.L., Heinrich M., Muñoz E. (2004). Imperatorin inhibits T-cell proliferation by targeting the transcription factor NFAT. Planta Med..

[29] Kowalczyk J., Nakos-Bimpos M., Polissidis A., Dalla C., Kokras N., Skalicka-Wozniak K., Budzynska B. (2021). Xanthotoxin affects depression-related behavior and neurotransmitters content in a sex-dependent manner in mice. Behav. Brain Res..

[30] Kokras N., Antoniou K., Mikail H.G., Kafetzopoulos V., Papadopoulou-Daifoti Z., Dalla C. (2015). Forced swim test: What about females?. Neuropharmacology.

[31] Kokras N., Dalla C., Papadopoulou-Daifoti Z. (2011). Sex differences in pharmacokinetics of antidepressants. Expert Opin. Drug Metab. Toxicol..

[32] Mikail H.G., Dalla C., Kokras N., Kafetzopoulos V., Papadopoulou-Daifoti Z. (2012). Sertraline behavioral response associates closer and dose-dependently with cortical rather than hippocampal serotonergic activity in the rat forced swim stress. Physiol. Behav..

[33] Barton D.A., Esler M.D., Dawood T., Lambert E.A., Haikerwal D., Brenchley C., Socratous F., Hastings J., Guo L., Wiesner G. (2008). Elevated Brain Serotonin Turnover in Patients With Depression: Effect of Genotype and Therapy. Arch. Gen. Psychiatry.

[34] Leopoldo M., Lacivita E., Berardi F., Perrone R., Hedlund P.B. (2011). Serotonin 5-HT7 receptor agents: Structure-activity relationships and potential therapeutic applications in central nervous system disorders. Pharmacol. Ther..

[35] Delgado P.L. (2000). Depression: The case for a monoamine deficiency. J. Clin. Psychiatry.

[36] Matos M.J., Vilar S., Vazquez-Rodriguez S., Kachler S., Klotz K.N., Buccioni M., Delogu G., Santana L., Uriarte E., Borges F. (2020). Structure-Based Optimization of Coumarin hA3 Adenosine Receptor Antagonists. J. Med. Chem..

[37] Yu-Taeger L., Ott T., Bonsi P., Tomczak C., Wassouf Z., Martella G., Sciamanna G., Imbriani P., Ponterio G., Tassone A. (2020). Impaired dopamine- and adenosine-mediated signaling and plasticity in a novel rodent model for DYT25 dystonia. Neurobiol. Dis..

[38] López-Cruz L., Salamone J.D., Correa M. (2018). Caffeine and Selective Adenosine Receptor Antagonists as New Therapeutic Tools for the Motivational Symptoms of Depression. Front. Pharmacol..

[39] Kokras N., Baltas D., Theocharis F., Dalla C. (2017). Kinoscope: An Open-Source Computer Program for Behavioral Pharmacologists. Front. Behav. Neurosci..

[40] Polissidis A., Koronaiou M., Kollia V., Koronaiou E., Nakos-Bimpos M., Bogiongko M., Vrettou S., Karali K., Casadei N., Riess O. (2021). Psychosis-Like Behavior and Hyperdopaminergic Dysregulation in Human α-Synuclein BAC Transgenic Rats. Mov. Disord..

[41] Kokras N., Pastromas N., Papasava D., de Bournonville C., Cornil C.A., Dalla C. (2018). Sex differences in behavioral and neurochemical effects of gonadectomy and aromatase inhibition in rats. Psychoneuroendocrinology.

[42] Kokras N., Antoniou K., Dalla C., Bekris S., Xagoraris M., Ovestreet D.H., Papadopoulou-Daifoti Z. (2009). Sex-related differential response to clomipramine treatment in a rat model of depression. J. Psychopharmacol..

[43] Novais A., Ferreira A.C., Marques F., Pêgo J.M., Cerqueira J.J., David-Pereira A., Campos F.L., Dalla C., Kokras N., Sousa N. (2013). Neudesin is involved in anxiety behavior: Structural and neurochemical correlates. Front. Behav. Neurosci..

